# Modeled Tradeoffs between Developed Land Protection and Tidal Habitat Maintenance during Rising Sea Levels

**DOI:** 10.1371/journal.pone.0164875

**Published:** 2016-10-27

**Authors:** Daniel Cadol, Andrew J. Elmore, Steven M. Guinn, Katharina A. M. Engelhardt, Geoffrey Sanders

**Affiliations:** 1 Earth and Environmental Science, New Mexico Institute of Mining and Technology, Socorro, NM, 87801, United States of America; 2 Appalachian Laboratory, University of Maryland Center for Environmental Science, Frostburg, MD, 21532, United States of America; 3 Center for Urban Ecology, National Park Service, Washington, DC, United States of America; University of Sydney, AUSTRALIA

## Abstract

Tidal habitats host a diversity of species and provide hydrological services such as shoreline protection and nutrient attenuation. Accretion of sediment and biomass enables tidal marshes and swamps to grow vertically, providing a degree of resilience to rising sea levels. Even if accelerating sea level rise overcomes this vertical resilience, tidal habitats have the potential to migrate inland as they continue to occupy land that falls within the new tide range elevations. The existence of developed land inland of tidal habitats, however, may prevent this migration as efforts are often made to dyke and protect developments. To test the importance of inland migration to maintaining tidal habitat abundance under a range of potential rates of sea level rise, we developed a spatially explicit elevation tracking and habitat switching model, dubbed the Marsh Accretion and Inundation Model (MAIM), which incorporates elevation-dependent net land surface elevation gain functions. We applied the model to the metropolitan Washington, DC region, finding that the abundance of small National Park Service units and other public open space along the tidal Potomac River system provides a refuge to which tidal habitats may retreat to maintain total habitat area even under moderate sea level rise scenarios (0.7 m and 1.1 m rise by 2100). Under a severe sea level rise scenario associated with ice sheet collapse (1.7 m by 2100) habitat area is maintained only if no development is protected from rising water. If all existing development is protected, then 5%, 10%, and 40% of the total tidal habitat area is lost by 2100 for the three sea level rise scenarios tested.

## Introduction

Tidal marshes occur in a fixed band of elevations, controlled by the local mean sea level and range of tides [1]. If mean sea level rises, marshes may migrate landward to higher ground or accumulate material and grow vertically to maintain elevation within the tidal frame; otherwise tidal marsh lands would be expected to transition into deeper water habitat classes, such as tidal flats, with associated loss of ecosystem services [2]. Marsh surface elevation change is affected by processes which increase elevation–such as mineral sediment deposition, organic matter deposition, and root growth–and those that decrease elevation–such as sediment compaction, organic matter decay, erosion, and local or regional subsidence [3–8]. Several of these processes depend on the elevation of the marsh relative to mean sea level, leading to both positive and negative feedbacks on relative marsh elevation change [9–11]. Additionally, sea level change, including both global eustatic components and regional gravitational or circulation effects [12], alters the relative elevation of a marsh surface.

In periods of rapidly rising sea levels, such as those forecast for the coming decades [13–15], marsh surface elevation gains may be overwhelmed by sea level rise (SLR) [16]. In this case, impediments to landward migration such as bluffs or levees will contribute to a reduction of marsh area and associated ecosystem services [17]. This effect is expected to be particularly prevalent because many engineering solutions that protect infrastructure from rising sea levels create just such a fixed landscape [18]. Undeveloped coastal areas, including managed open space and agricultural land, are less likely to be protected from SLR, and may be crucial refuges to which marshes may migrate.

**Fig 1:**
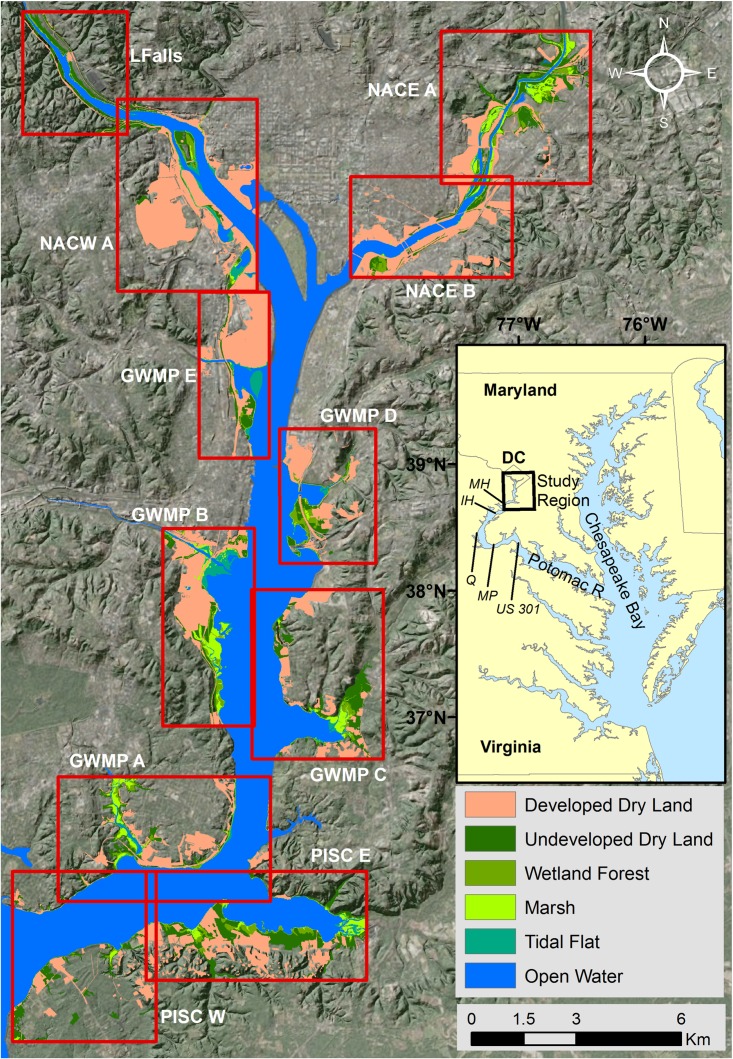
Study area map. The eleven sub-regions are outlined in red boxes and labeled in white. Geographic features in the sub-regions include: PISC W- Marshall Hall; PISC E- Mockly Point and Piscataway Creek; GWMP A- Little Hunting Creek and Mount Vernon; GWMP B- Dyke Marsh; GWMP C- Broad Creek; GWMP D- Oxon Creek; GWMP E- Dangerfield Island and Reagan National Airport; NACE B- Anacostia Park; NACE A- Kingman Lake and Kenilworth Marsh; NACW A- Theodore Roosevelt Island and Columbia Island; LFalls- Chain Bridge and Little Falls. Black italic labels in the location map identify significant points on the Potomac River downstream of the study area: MH- Marshall Hall; IH- Indian Head; Q- Quantico; MP- Maryland Point; and US 301- US Highway 301 Bridge.

The strategic management of coastal open space benefits from marsh migration forecasts [19]. Models that track habitat class as a function of relative elevation (e.g. the Sea Level Affecting Marshes Model, SLAMM, [20]) have been effectively employed to this end. Most attention, however, has been focused on salt marshes [20, 21] or large deltaic systems that cross from fresh to brackish habitats [19, 22–24]. Frequently these models are applied over very large areas at relatively coarse grids (30 m to 1 km cells). Without downplaying the importance of saltwater systems, the research presented here seeks to extend this work to estuarine freshwater tidal marshes. Freshwater marshes occur in most estuarine systems, where tidal action extends upstream along large river courses. Though they are often smaller than salt marshes, estuarine freshwater marshes are important ecosystem components, contributing disproportionately to nutrient retention and providing additional ecosystem services including flood control and habitat for many rare, threatened, and endangered species [25–28]. The work presented here was conducted along the tidal Potomac River, where numerous National Park Service (NPS) management units preserve open space in an elevation range relevant to marsh migration within the next century (Fig 1). Because this work was conducted in a freshwater tidal environment, SLAMM had some features that were not ideal (e.g., implicit salinity effects), and lacked other features that we deemed important (e.g., gradational vegetation zonation). From an ecological standpoint, salt marshes have both an anoxic stress gradient (due to inundation time) and a salinity gradient (due to evaporation, especially in warm environments), which together contribute to highly distinct zonation of species [1]. Freshwater marshes only have the anoxic stress gradient, and individual species are more loosely zoned, with a great deal of elevational overlap between species [29].

Therefore we developed and tested a spatially explicit elevation-tracking and vegetation class-switching model, which we designated Marsh Accretion and Inundation Model (MAIM). Effort was made to develop a parsimonious model that also adequately captures elevation gain processes. Other morphodynamic models have produced good results at similar scales [16, 30], but these require data on vegetation biomass distribution, sediment trapping efficiency, and other highly variable parameters. MAIM utilizes a consolidated, empirical elevation gain function to represent the biogeomorphic feedbacks that drive marsh elevation change in each vegetation class. Simplified process modeling or reduced-complexity modeling such as this attempts to optimize the trade-off between increased fidelity to physical processes and increased uncertainty in the model parameters that describe these processes [31, 32].

Our implementation used control parameters calibrated for the freshwater tidal river reach of the Potomac estuary, but to promote continued use in dissimilar systems MAIM was intentionally programmed with a high degree of flexibility, allowing for changes in the number of habitat classes, their sequencing, their elevation ranges, and their elevation gain functions. In this paper we briefly describe the model mechanics and calibration, and then present model results for three SLR scenarios crossed with two ‘development protection’ scenarios for which MAIM was initially run. These six model runs enable an analysis of the interacting effects of potential SLR and development protection on marsh area loss.

## Methods

### Study Area

We modeled 11 sub-regions of the upper tidal Potomac River system, from Mount Vernon and Piscataway Park (38°41’ N, 77°06’ W) at the downstream to Little Falls on the Potomac (38°56’ N, 77°07’ W) and Kenilworth Marsh on the Anacostia River (38°55’ N, 76°57’ W) at the upstream (Fig 1). The sub-regions ranged in size from 10 to 20 km^2^, however only water and land below 5 m elevation (NAVD88) was modeled, resulting in a total area of 6,925 ha. Of this 4,140 ha started as estuarine open water and 1,315 ha was classified as developed. The region is a highly fragmented patchwork of forest, agriculture, and developed land and contains a variety of small NPS units that protect cultural and historic sites as well as natural resources including freshwater tidal marshes, swamps, and adjacent lands. The disaggregated nature of the managed natural spaces creates management challenges, but their relative abundance within an urban area also creates opportunities for innovative management approaches.

### Model Overview

MAIM requires six inputs: a high-resolution DEM, an initial land classification map, elevation limits for each land class described as probability density functions, land class switching rules, surface elevation change functions for each land class, and a SLR scenario. At each time step MAIM (1) alters the surface elevation of each cell according to the elevation change rules provided, (2) changes sea level according to the SLR scenario provided, (3) determines if the relative elevation of any cells have left the range assigned to the land class of that cell, and if so, (4) switches the land class of those cells according to the user defined rules. Land class elevation limits are picked randomly for each model run from user defined probability density functions. We designed the model to be run repeatedly in a Monte Carlo experiment, with the most probable class of each cell and the probability of obtaining this class calculated at pre-selected output times. Outputs for individual model runs are a DEM and a land classification raster. Outputs for Monte Carlo suites of runs also include a majority (or plurality) land classification raster, a majority class probability raster, and summary statistics.

### Model Inputs

#### Elevation data

For elevation we used an airborne LiDAR-derived DEM with 1 m cells and 1 cm vertical precision (~ 2 cm vertical accuracy on hard surfaces), which was provided by the NPS National Capital Region GIS Office. The LiDAR survey was acquired between 10/31/2008 and 12/23/2008. The raw data are one meter posting, first and last return, and were used prior to this project by technicians at Science Applications International Corporation to produce a 1-meter bare earth DEM. Validation of the DEM is described in supporting section S1 File and S1 Fig.

#### Land classes

MAIM can accept any combination of user-defined land classes. For this application we included fourteen classes, which were mapped using the LiDAR derived DEM, an NPS-commissioned vegetation classification polygon layer [33], a heads-up digitized (HUD) forest cover mask from National Agriculture Imagery Program (NAIP) imagery, a HUD open water mask (representing the transition from tidal flat to open water), a weighted distance from water map, and a HUD developed land mask. The mapping procedure is described in supporting section S2 File and S2 Fig. The validation of these maps using ground truth points is described in supporting section S3 File and S1 Table.

The fourteen land classes were Non-NPS Developed Dry Land, NPS Developed Dry Land, Managed Open Space, Agriculture, Undeveloped Dry Land, Undeveloped Dry Land With Swamp Species, Wetland Forest, Irregularly Flooded Forest, Transitional Scrub, Ephemerally Flooded Marsh, Irregularly Flooded Marsh, Regularly Flooded Marsh, Tidal Flat, and Estuarine Open Water (Table 1). The first four classes were considered developed land, though the development ranged from hard (e.g., pavement, buildings) to soft (e.g., parks, golf courses). The only agriculture in our area was the colonial era demonstration farm at Piscataway National Park. These developed classes were mapped at all elevations, and were given no lower elevation limit in the scenarios that protected development.

**Table 1:** Land class characteristics.

Land Class	Characteristics ^a^	Elevation Limits ^b^	Typical Inundation
Developed Dry Land (non-NPS)	hard development (e.g., pavement, buildings) with some associated landscaping	none or >2.1 HTU	none
Developed Dry Land (NPS)	hard development on NPS managed land	none or >2.1 HTU	none
Managed Open Space	soft development (e.g., pasture, open space, parks, and golf courses)	none or >2.1 HTU	none
Agriculture	crop fields (primarily Piscataway Colonial Demonstration Farm in our study area)	none or >2.1 HTU	none
Undeveloped Dry Land	floodplain forest, successional deciduous forest, and mesic mixed hardwood	>2.1 HTU	none
Undeveloped Dry Land with Swamp Species	Upland forests that had some presence of Sweetgum (*Liquidambar styraciflua*), Red Maple (*Acer rubrum*), Pumpkin Ash (*Fraxinus profunda*), Green Ash (*Fraxinus pennsylvanica*), or Swamp Blackgum (*Nyssa biflora*)	>2.1 HTU	none
Wetland Forest	maple-ash swamp and some oak floodplain swamp; inundated by tidal events above the typical spring high high water	1.3 to 2.1 HTU	monthly to annually
Irregularly Flooded Forest	tidal swamps such as swamp blackgum-ash and sweetgum-tuliptree temporarily flooded forests	0.7–1.2 HTU	0–6 hours most days
Transitional Scrub	disturbed woody wetland, successional herbaceous wetland, vineland, shrubland, and all marsh lands higher than 2.1 HTUs; in spite of rare inundation, a hydrological influence from tides is expected.	variable; mostly 1.9–2.1 HTU	rarely; large storms or floods
Ephemerally Flooded Marsh	highest marsh elevations; *Typha* sp. dominant, but included some dogwood stands	1.7–1.9 HTU	several times per year
Irregularly Flooded Marsh	dominated by *Typha* sp., *Schoenoplectus* sp., and *Impatiens* sp., with locally dominant patches of *Acorus* sp. and *Peltandra* sp.	0.5–1.7 HTU	0–6 hours most days
Regularly Flooded Marsh	dominated by *Nuphar* sp. and *Peltandra* sp.	-0.5–0.5 HTU	6–18 hours most days
Tidal Flat	may be vegetated with submerged aquatic vegetation (SAV) or may be barren mud flat	-1.3 to -0.5 HTU	24 hours most days
Estuarine Open Water	open water	< -1.3 HTU	permanently inundated

^a^ Forest vegetation characteristics are based on an NPS-commissioned vegetation classification map.

^b^ Elevation limits are given in half tide units (HTUs) and are the means of the distributions from which model values were drawn. Lower limit of developed categories depends on the development protection scenario.

**Fig 2:**
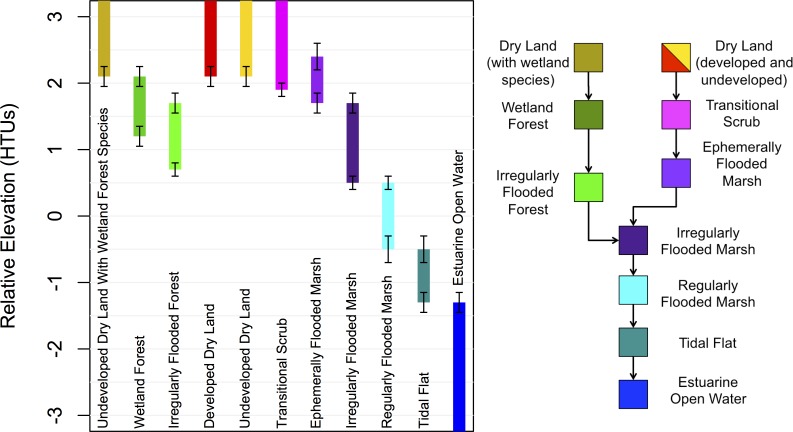
Elevation limits of land cover classes, and class switching flow chart. Whiskers on elevation ranges indicate one standard deviation of the Gaussian distribution from which habitat transition elevations were selected in the repeated Monte Carol runs.

Undeveloped dry land (and the developed classes in the unprotected scenarios) had a lower elevation limit of 2.1 half tide units (HTUs) or approximately 1 m absolute elevation (all absolute elevations are reported in the NAVD88 datum). Half tide units (as used in SLAMM) record elevation relative to mean sea level (MSL), and each unit is half of the range between the mean higher high water (MHHW) and mean lower low water (MLLW). Assuming symmetric high and low tides, MSL will have an elevation of 0 HTU, MHHW will be at an elevation of 1 HTU, and MLLW will be at −1 HTU. Thus the lower limit of dry land was just over twice the elevation of MHHW, an elevation for which there was no record of tidal flooding for at least eight years preceding the study [34]. When SLR caused dry land cells to drop below this lower relative elevation limit, they either entered a marsh habitat track or a swamp forest habitat track (Fig 2). To decide, we defined the Undeveloped Dry Land With Swamp Species category, which identifies upland cells that, according to the NPS vegetation maps, contained tree species characteristic of swamp forests that might persist if the land started to experience tidal flooding (Table 1; e.g., *Acer rubrum*, *Fraxinus profunda*, *Nyssa biflora*, etc).

Both MSL and the tide range varied among our modeling sub-regions, with MSL increasing upstream due to fluvial influence and tide range increasing upstream due to narrowing of the estuary. According to the National Oceanic and Atmospheric Administration data that we used in our model runs, MSL increased from an absolute elevation of 0.029 m at the lowermost site (Piscataway) to 0.048 m at the uppermost site (Little Falls) for the 1983–2001 epoch. Tide range increased from 0.802 m to 0.952 m at these same sites. Converting HTU elevations to absolute elevations at Piscataway, for example, −1 HTU was equivalent to −0.372 m elevation, 0 HTU was equivalent to 0.029 m, and 1 HTU was equivalent to 0.430 m.

#### Class switching rules

All class-switching rules in MAIM are user defined. In our implementation, rising sea level caused cells to switch classes when relative elevation dropped below the lower limit for their current class. Each class was assigned an upper and lower elevation limit (Fig 2). Because the initial land class maps were not just elevation-based, leading to potential inconsistencies with these user defined elevation limits, the first modeling step was a time zero (t_0_) correction. This correction step also applied a small amount of sea level rise to account for the fact that the initial land classification map was developed with 2008 data, and the first model time was 2010. This was the only modeling step where a cell may convert to a higher elevation class. Otherwise cell switching follows unidirectional class-dependent rules (Fig 2). Because our implementation of MAIM assumes only rising relative sea level, cells identified as Estuarine Open Water could not transition into anything else. In addition to the base switching rules, we also created a second rule set that protected all development (hence two ‘protection scenarios’). This was accomplished by eliminating the lower elevation limit of the Developed Dry Land (including NPS land), Managed Open Space, and Agricultural classes to prevent them from ever switching.

The upper and lower elevation limits of each land class are an important source of uncertainty in our model results. We do not, in fact, expect the actual landscape to follow such strict elevation rules. Therefore we attempted to model uncertainty by allowing the elevation limits to vary between runs. Each model scenario (SLR and protection scenario combination) was run 100 times, with elevation limits picked randomly from Gaussian distributions centered on the nominal limits (Table 1). The range of one standard deviation for each boundary is shown in Fig 2. These estimates of standard deviation were based on differences between the initial classification map and field observations at three validation sites (S1 Table). In cases where class transitions are controlled by elevation the lower boundary value of one class must match the upper boundary of another class, and in such cases they were varied together.

#### Elevation change module

**Fig 3:**
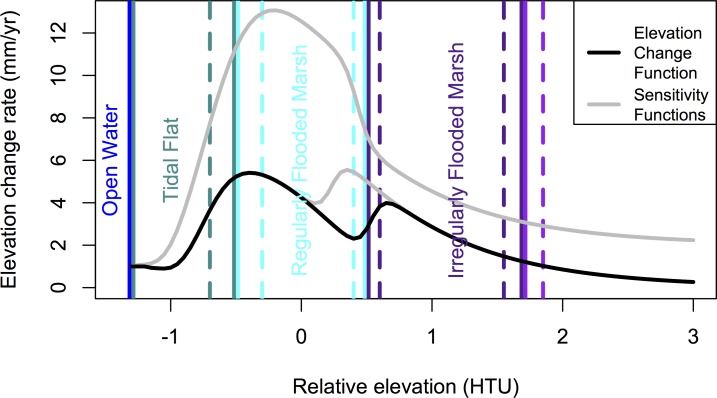
Elevation gain vs. elevation functions. The empirical function utilized for marsh habitat elevation gain in the simulations is shown in black (Eq 1), and two alternative elevation-gain functions that were used to test model sensitivity to uncertainty in elevation gain rates are shown in gray. Vertical lines show habitat zonation, with dashed lines indicating one standard deviation of the Gaussian distribution from which habitat transition elevations were selected.

MAIM changes the land surface elevation at each time step as a representation of net accretion, subsidence, and erosion. Any elevation change (Δ*E*) function that depends on elevation (*E*) can be applied in the code to each land class type (i.e. Δ*E* = *f*(*E*, class)). Our implementation of MAIM does not attempt to model time-variant erosion or shoreline erosion. Earlier work in Dyke Marsh, one of the tidal marshes in the study area, led to the development of the elevation change function utilized for marsh habitats [11], (Fig 3):

$$\Delta E=S_{n}e^{-\frac{{\left[\ln\left(E-m_{n}\right)\right]}^{2}}{2\sigma_{n}^{2}}}+S_{l}\left[\frac{1}{1+e^{-k_{l}\left(E-m_{l}\right)}}-1\right]+S_{u}\left[\frac{1}{1+e^{-k_{u}\left(E-m_{u}\right)}}-1\right]$$ (1)

where the first term on the right is a log-normal function, and the second and third terms are logistic sigmoidal functions that vertically (Δ*E*-dimension) shift the base log-normal function in two zones. This mathematical form was selected as a parsimonious method to fit observed trends, and parameter values were selected to minimize residuals. This equation is likely over-parameterized, and we do not suggest particular ecogeomorphic meanings for the values, but the parameters were retained because each may have meaning. The three *S* parameters are scaling values for each of the sub-functions. The *m* parameters laterally shift (*E*-dimension) the sub-functions, defining the midpoints of the sigmoids and mean of the log-normal. The *k* terms control the spread of the two sigmoids, and *σ*_*n*_ is the standard deviation of the log-normal distribution, which controls its spread. The log-normal function is undefined if *E* ≤ *m*_*n*_, in which case the accretion rate for tidal flats was applied. The parameter values used to match elevation change in the Dyke Marsh calibration area are presented in Table 2.

**Table 2:** Elevation change function (Eq 1) parameter values used.

Log-Normal Curve		Lower Sigmoid		Upper Sigmoid	
Parameter	Value	Parameter	Value	Parameter	Value
σ_n_	0.55	k_l_	6.5	k_u_	20
S_n_	0.008	S_l_	0.0045	S_u_	-0.0035
m_n_	-1.5	m_l_	-0.8	m_u_	0.23

Our elevation change function was derived by differencing two elevation surveys of Dyke Marsh, one conducted in 1992 [35] and one in 2012 [11]. Systematic variation in elevation change over these 20 years was observed as a function of original elevation, which is represented in Eq 1 (Fig 3), but there was a broad range of variability. Eq 1 gives the best-fit mean elevation change rate for a given elevation, but the 95% confidence interval for this mean behavior extends ± 2 mm/yr above and below the estimate, and the 95% prediction interval for individual points extends ± 5 mm/yr. The variability incorporates spatially discontinuous processes that occurred over the 20 years between surveys, e.g., blow downs and storm overwash related erosion, but it does not include tidal creek migration or shoreline erosion, as points affected by these processes were excluded from the analysis. Because Eq 1 empirically incorporates all processes that affected elevation of the marsh interior from 1992 to 2012, any future changes such as altered storm frequency and related overwash, changes in nutrient or sediment delivery, or invasion of new species would affect the reliability of this function.

The peak of our elevation change function is 6 mm/yr, occurring at ~ −0.4 HTU (~ −0.2 m elevation), with a secondary peak of 4 mm/yr at ~ 0.6 HTU. This peak limits resilience to SLR; any SLR rate greater than the peak will eventually lead to inundation, an important consideration given the range of rates we modeled (4 to 9 mm/yr for the lowest scenario and 10 to 21 mm/yr for the highest; Table 3). Some marsh points gained elevation at a greater rate, but the field data suggest that if recent elevation gain rates persist then there is a 95% probability that the limiting elevation gain rate is between 4–8 mm/yr in Dyke Marsh [11], and by extrapolation the full study region. Suspended sediment supply has been shown to be a major control on accretion rates [36, 37], so changes relative to the 1992–2012 period would necessitate modification of the function. Suspended sediment concentration in the Potomac River at Chain Bridge, the upstream limit of our uppermost sub-region at Little Falls (Fig 1), from 1985–1996 had a mean of 18 mg/L and a median of 108 mg/L, with large flood events producing concentrations as high as 2,990 mg/L [38]. Annual suspended sediment loads from 1985 to 2012 ranged from 0.3 to 5.8 million metric tons (mean 1.8 10^6^ metric tons) though no trend is discernable [38, 39]. Tile sedimentation studies at Dyke Marsh suggest that the total suspended sediment load of the Potomac may be a better predictor of accretion than suspended sediment concentration [40].

**Table 3:** Sea level rise scenarios.

	0.7 m Scenario		1.1 m Scenario		1.7 m Scenario	
Year	Sea level (m)	SLR rate (mm/yr)	Sea level (m)	SLR rate (mm/yr)	Sea level (m)	SLR rate (mm/yr)
2010	0.06		0.1		0.15	
2020	0.1	4	0.16	6	0.25	10
2030	0.15	5	0.24	8	0.37	12
2040	0.22	7	0.34	10	0.52	15
2050	0.29	7	0.45	11	0.7	18
2060	0.36	7	0.57	12	0.88	18
2070	0.45	9	0.7	13	1.08	20
2080	0.53	8	0.84	14	1.29	21
2090	0.62	9	0.97	13	1.5	21
2100	0.7	8	1.1	13	1.7	20

We applied Eq 1 to Ephemerally, Irregularly, and Regularly Flooded Marsh categories, which permits a smooth transition in elevation change across these boundaries (Fig 3). The elevation change characteristics of Dyke Marsh [11] were assumed to be representative of all marshes in the study region. Surface elevation change rates for wetland forests, tidal forests, and tidal flats were given fixed accretion values due to the paucity of data relating elevation change to elevation in these habitat types [41, 42]. We elected to use elevation gain rates of 0.0003 m/yr for wetland forests and 0.0011 m/yr for tidal forests (after [41]), and 0.001 m/yr for tidal flats. These values are highly uncertain, but we do expect accretion rates in these areas to be smaller than in nearby marshes [42]. If the elevation gain rates used in the model are lower than reality, then the results will overestimate area loss in these tidal habitat types. No elevation change was applied to dry land categories, though their relative elevation did change as sea level rose.

#### Sea level rise scenarios

Three SLR scenarios were adopted from work by the Scientific and Technical Working Group of the Maryland Climate Change Commission (MCCC; [43]). The MCCC reported that projections provided by the Intergovernmental Panel on Climate Change (IPCC) 4th Assessment Report were too conservative in that they did not include flows from the polar ice sheets, an evaluation consistent with other recent findings [13] and which is addressed in the IPCC 5^th^ Assessment Report [14]. Further, the MCCC report accounted for local variation in SLR due to regional ocean dynamics and land subsidence. The MCCC did not account for the potential for a weakening of the Gulf Stream, which could raise local sea levels significantly (> 0.20 cm) in the next decade [44]. The MCCC estimates were not reported as an annual time series of SLR suitable for direct input to MAIM, but rather as low, medium, and high projections of SLR to 2050 and 2100 (Table 3). We therefore incorporated temporal trends from the IPCC 5th Assessment Report RCP 6.0 scenario, utilizing the IPCC curve shape but scaling the 2100 value to match the MCCC estimates of total accumulated SLR through 2100, which were 0.7, 1.1, and 1.7 m (Table 3). The curve shape is likely consistent between the two assessments because the scaled 2050 SLR values matched the MCCC projections within 2 cm. The highest MCCC projection includes 0.75 m of SLR related to rapid collapse of the Greenland and Antarctic ice sheets, a poorly constrained process [45, 46]. The MCCC projections are consistent with US Army Corps of Engineers guidance [47] and a National Research Council report for the Pacific Coast of the United States [48]. But other regional forecasts of SLR to 2100 extend above and below the range projected by the MCCC [49]. At the low end of the range, the IPCC 5AR projects global SLR increases of 0.28–0.61 m by 2100 for a scenario in which CO_2_ emissions rapidly cease (RPC2.6) [14]. The MCCC report estimates 0.13–0.17 m of relative SLR due to land subsidence, and 0.13–0.19 m of SLR due to changes in regional ocean dynamics (not including changes to the Gulf Stream), suggesting the possibility of local relative SLR as low as 0.54 m. For comparison, the historic SLR trend at Washington, DC is 3.2 mm/yr for the period 1924–2015, which when compared to global eustatic SLR of 1.7 mm/yr suggests regional subsidence of 1.5 mm/yr. Given the uncertainty regarding many processes driving SLR and the range of values possible, we do not intend to suggest that the 1.1 m scenario is the most likely due to its central position in the three scenarios we tested.

## Results

### Initial land class maps

**Fig 4:**
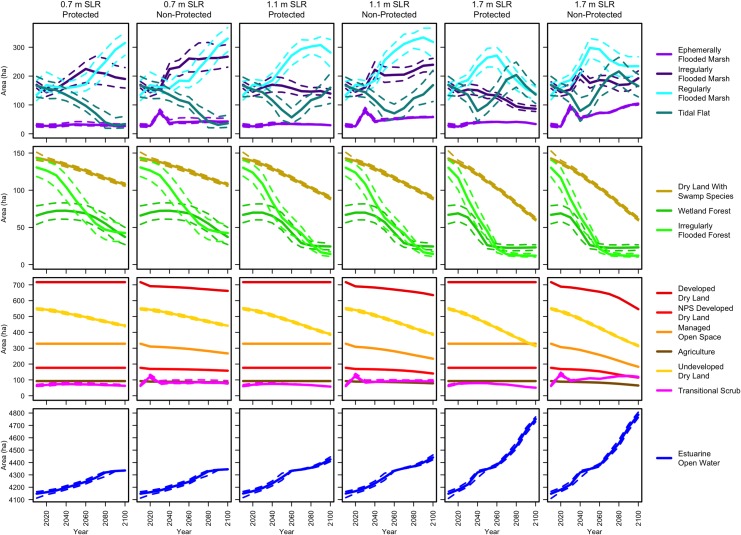
Habitat area through time for the 6 modeled scenarios. Solid lines are the median values of the 100 Monte Carlo runs, and the dashed lines are the 1^st^ and 3^rd^ quartiles.

The magnitude of the zero time (t_0_) correction that was applied to the initial land classification maps can be thought of as an indicator of the degree of agreement between our initial land classification assumptions and our elevation limit estimates. Because the elevation limits were selected stochastically for each model run from a defined distribution, the amount of change varied. For the case using the mean values of the elevation limits, the largest changes, or assumption disagreements, were for wetland forest (+39 ha; +105%), irregularly flooded forest (−71 ha; −37%), irregularly flooded marsh (+51 ha; 38%), tidal flats (−35 ha; −18%), regularly flooded marsh (+13 ha; +9%), and ephemerally flooded marsh (+8 ha; +27%), with other classes changing 3% or less (S2 Table). The majority of these changes are irregularly flooded forest being moved up to wetland forest, or down to a marsh class, a change that is consistent with our reliance on inputs other than elevation, such as weighted distance to water and manually digitized forest, in classifying tidal and wetland forests (S2 Fig). This extra information is excluded in the habitat switching rules of MAIM, which may result in less reliable mapping of swamp forest categories. One exception in the application of the t_0_ correction was for the developed land classes. Development was not converted to any other class until the first time step, 2020. As a result, for the unprotected development scenarios, there is a mass of land area that goes from developed land in 2010 to transitional scrub in 2020 to ephemerally flooded marsh in 2030 and on down the sequence (Fig 4). This migrating spike is an artifact of low-lying developed land only being permitted to drop one class per 10-year time step, but the artifact is eliminated by 2070.

### Land cover distribution

Modeled trends of habitat class coverage through time depended heavily on both SLR scenario and whether development was protected (Fig 4). The elevation dependent accretion rates applied to the marsh habitats contributed to complexity and divergence of results for the three SLR scenarios. For example, in the protected development scenarios, irregularly flooded marsh was able to maintain position in the tidal frame and maintain its aerial coverage in the 0.7 m SLR scenario, while it showed increasing losses for the 1.1 m and 1.7 m SLR scenarios. When development was allowed to convert to marsh, irregularly flooded marsh gained area in all three SLR scenarios. Regularly flooded marsh experienced even greater area gains, especially in the 0.7 m SLR scenario, because the peak of the elevation change function that fell within this class range was higher (Fig 3). But in the 1.1 m and 1.7 m SLR scenarios even regularly flooded marsh began loosing area after 2050 and 2070, respectively, with gains going to the tidal flat class (Fig 4). Swamp forests saw significant declines in all scenarios, though this may have been influenced by the uncertainty in selecting accretion rates for these habitats. Undeveloped dry land experienced the largest area losses of any land class, and irregularly flooded forest lost the largest percent of area. Significant proportions of development, managed open space, and agriculture converted to marsh habitats in the non-protection scenarios (25%, 35%, and 45% respectively in the 1.7 m SLR scenario; 8%, 10%, and 19% in the 0.7 m SLR scenario). Estuarine open water gained the most area of any category in all scenarios.

**Fig 5:**
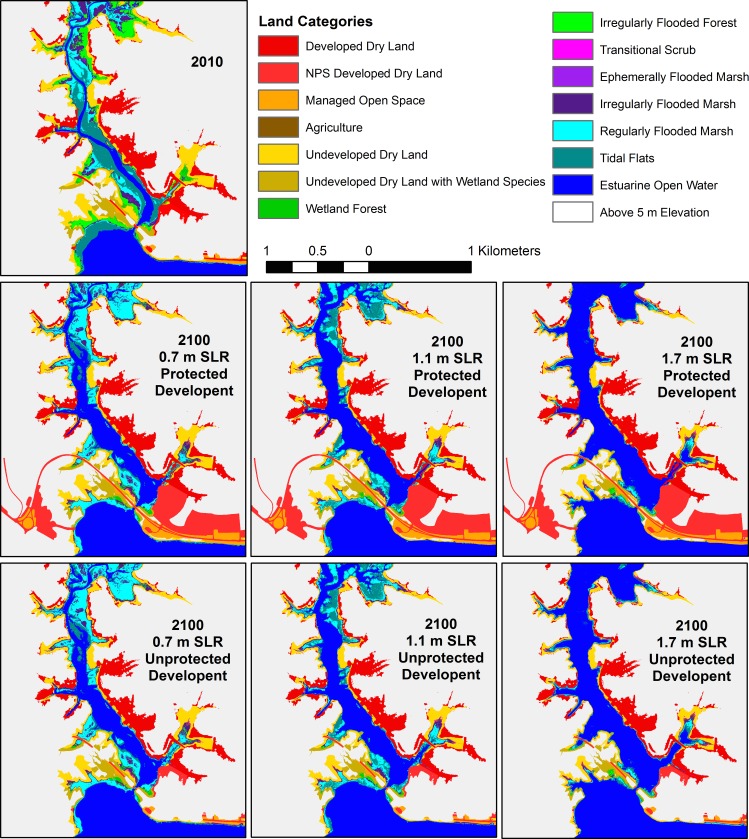
Model results for the Little Hunting Creek area (GWMP A). Beginning model land classifications for 2010 (following the initial time correction), and model results for the six scenarios.

**Fig 6:**
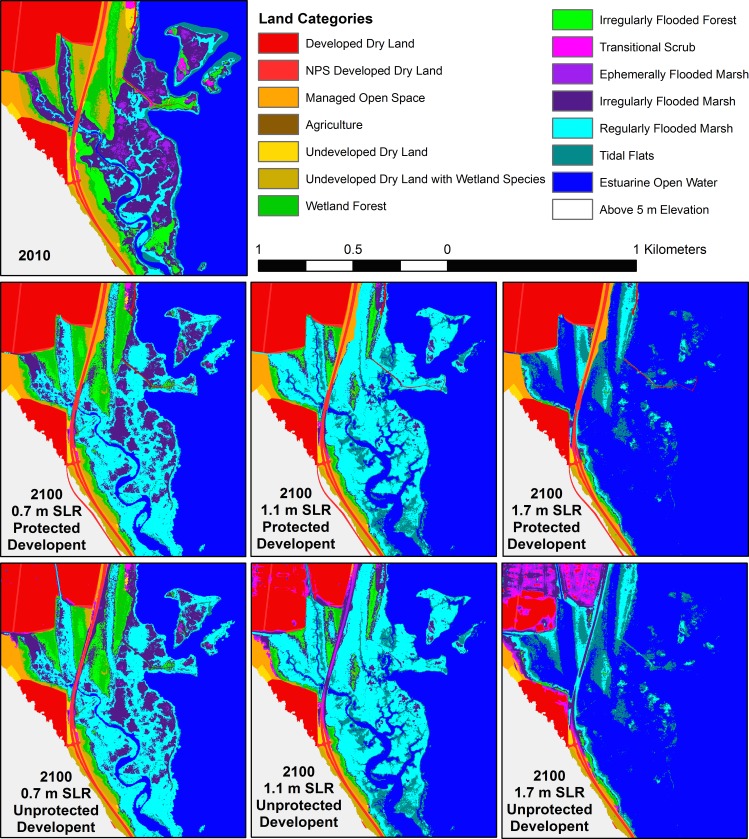
Model results for the Dyke Marsh area (GWMP B). Beginning model land classifications for 2010 (following the initial time correction), and model results for the six scenarios.

Land class evolution followed patterns related to the two drivers included in the model: topography and habitat type. In tidal habitats surrounded by steeply sloping valley walls, strong differences in pattern were observed in the three SLR scenarios. For example, in the Little Hunting Creek area of the GWMP A sub-region (Fig 5) strips of marsh habitat as narrow as a few meters wide were all that remained of the tidal habitat by 2100 in the highest 1.7 m SLR scenario. But in the 0.7 m SLR scenario, only tidal flats were lost to open water while marsh habitat persisted and expanded. The effect of lower modeled accretion rates in swamp habitats is observed in the conversion of these areas to tidal flat by 2100 in the 1.1 m SLR scenario, even as marshes remained marsh (Fig 5). In contrast, where slopes near the river were gentle, significant inland migration was observed. In the Dyke Marsh area of the GWMP B sub-region, swamps migrated inland and marshes persisted and expanded in the 0.7 m SLR scenario (Fig 6). Most swamp was converted to marsh, and nearly all marshland was restricted to regularly flooded marsh (low marsh) by 2100 in the 1.1 m SLR scenario. For the 1.7 m SLR scenario, the vast majority of the marsh was inundated and converted to open water, and if development was not protected a large swath of a housing development converted to high marsh by 2100 (Fig 6). Just north is a golf course that experienced similar conversion to marsh if not protected.

**Fig 7:**
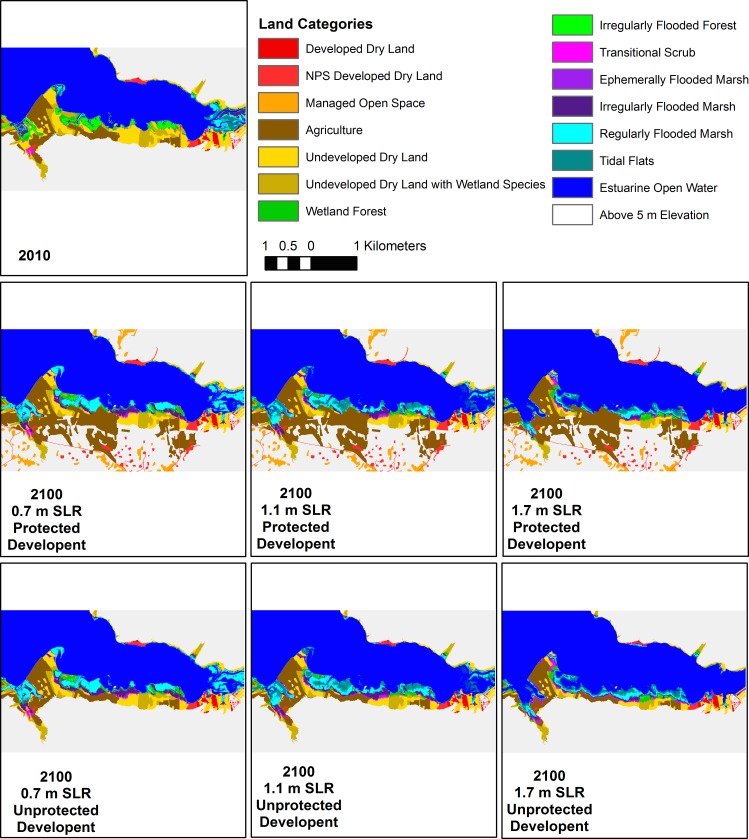
Model results for the Piscataway Park area (PISC E). Beginning model land classifications for 2010 (following the initial time correction), and model results for the six scenarios.

**Fig 8:**
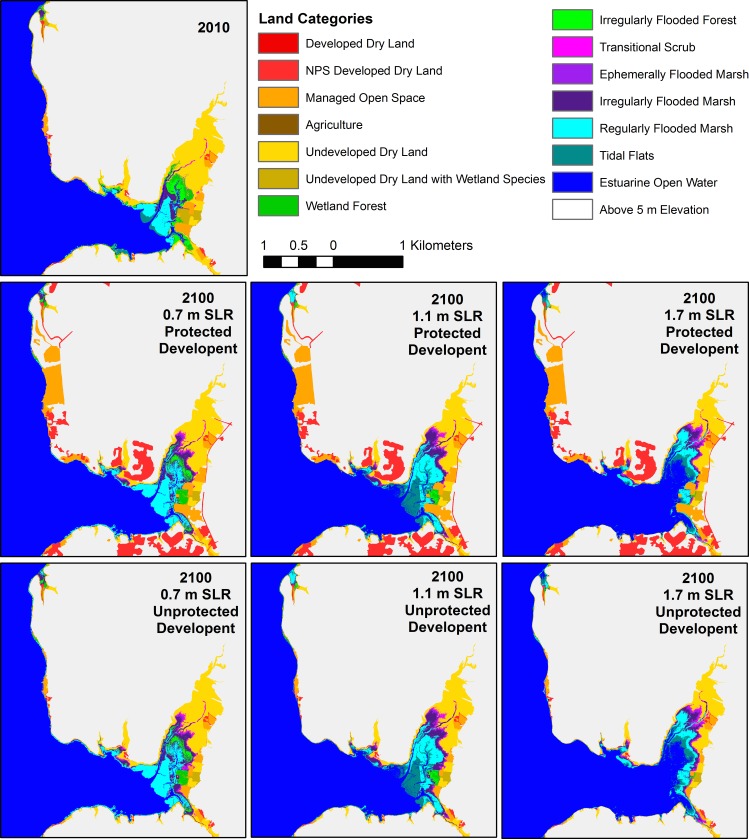
Model results for the Anacostia River/Kenilworth Marsh area (NACE A). Beginning model land classifications for 2010 (following the initial time correction), and model results for the six scenarios.

**Fig 9:**
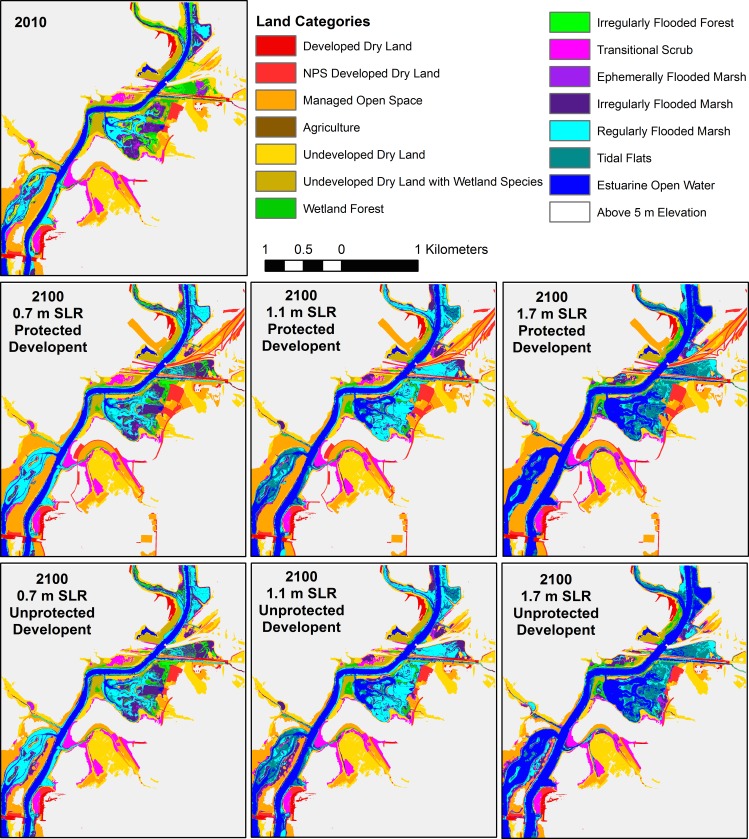
Model results for the Broad Creek area (GWMP C). Beginning model land classifications for 2010 (following the initial time correction), and model results for the six scenarios.

Landward marsh migration into soft development was key to marsh habitat maintenance in the Piscataway Park area, the only sub-region with agricultural land, especially in the 1.1 m and 1.7 m SLR scenarios (Fig 7). However expansion into managed open space was much less important in the upper Anacostia River, in spite of the fact that it is surrounded by development (Fig 8). The high banks limited dry land conversion to tidal habitat, even in the highest SLR scenario, and the greater tide range reduced the relative effect of SLR. The differing degrees of landward marsh migration with different SLR rates were clearly visible on the gently sloping surface of Broad Creek marsh (Fig 9). Marsh habitat patches remained relatively narrow swaths in this area in all scenarios, on the order of 10–50 m wide, but migrated inland greater distances in the 1.7 m SLR scenario.

### Effect of development protection

**Fig 10:**
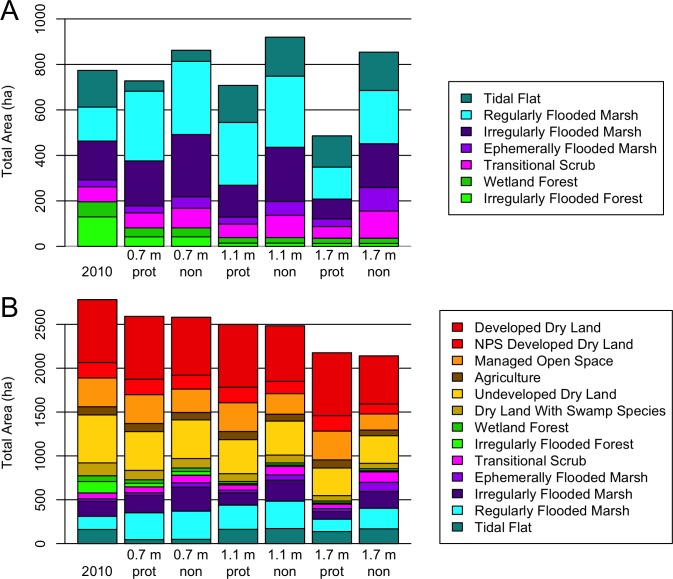
Year 2100 modeled habitat distributions. (A) Tidal habitats only. (B) All classes other than open water. Low, med, and high refer to the 0.7 m, 1.1 m, and 1.7 m SLR scenarios. Protected development scenarios (“prot”) and non-protected development scenarios (“non”) are plotted for each SLR scenario.

The model scenarios in which developed land was protected led to tidal habitat losses over the modeled period (2010–2100) while allowing developed land to convert to tidal habitat led to habitat area gains, even in the highest SLR scenario (Fig 10A). Tidal habitats here include categories from wetland forest and transitional scrub down to tidal flats. The greatest net gain of tidal habitat was for the 1.1 m SLR scenario with development not protected, and the greatest gain excluding tidal flats was for the 0.7 m SLR scenario with development not protected. If development was protected, then about 10% of the tidal habitat area was lost by 2060 in the 1.7 m SLR scenario, accelerating to 40% loss by 2100. If developed land was sacrificed, very little of that land became open water by 2100, even in the 1.7 m SLR scenario; it nearly all contributed to tidal habitat. Managed open space, agriculture, and NPS developed land made up 60% of the sacrificed land that converted to tidal habitat in the unprotected 1.7 m SLR scenario, while 40% was non-NPS hard development (Fig 10B). Thus according to the model results, net tidal habitat loss by 2100 (either excluding or including tidal flats in that group) could be avoided in a medium SLR scenario if soft development was permitted to be flooded, and habitat loss could even be avoided in a high SLR scenario if all development was unprotected.

### Elevation change

**Fig 11:**
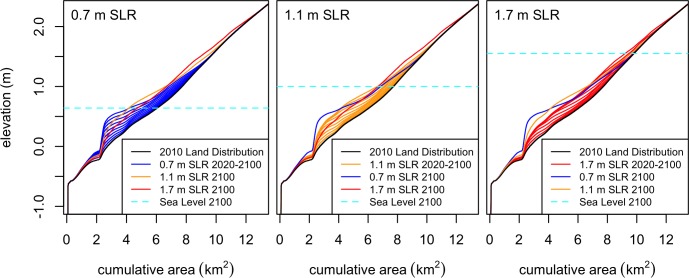
Hypsometric curves of the surface elevation distribution. Data covers the entire study region, and is separated among the 3 different sea level rise scenarios. Cumulative area (x-axis) is the area with elevations exceeded by the corresponding elevation (y-axis) along each curve.

Equivalent habitat changes occurred later in lower SLR scenarios, however the exact nature of the changes differed due to the interaction of elevation and elevation gain modeled in MAIM. The distribution of elevation gains varied greatly through time and among the SLR scenarios (Fig 11). The increasing open water gains in higher SLR scenarios are due to vertical land loss. Although total tidal habitat area was maintained to 2100 under 1.7 m SLR without protection, the resultant landscape had a smaller land area (less dry land, especially at elevations near the tide level) and would be less robust to continued SLR than the lower SLR scenarios.

## Discussion and Conclusions

The complex biogeomorphic interplay between SLR and marsh accretion has long been recognized, with local factors expected to cause great variation in response [50]. And as expected, some investigations of coastal response to SLR have forecast major habitat loss (e.g., [20]) while others predict minimal change (e.g., [51]). Estimating the threshold SLR in each case beyond which habitat loss accelerates can provide an appropriate metric for comparison among studies and help resolve conflicting interpretations [52]. Several of our model scenarios resulted in increased tidal habitat in 2100 relative to 2010. All three SLR scenarios yielded habitat gains when development was not protected. The key influence of development protection is in agreement with previous modeling work on the Gulf Coast of Texas [21]. The resilience in our study also hinged on the elevation gain rates, particularly the maximum gain rate in regularly flooded marshes (Fig 3), though the local maximum in irregularly flooded marshes was important in the partitioning of land between these two classes.

Tidal habitat gains were derived from a combination of elevation gains preserving existing marsh and inundation converting dry land to marsh or swamp. The land inundation is well controlled, although uncertainty remains regarding whether it will become marsh or swamp. On the other hand, marsh accretion rates are calibrated at a single area (Dyke Marsh; [11]) and tidal flat and tidal forest rates are estimates from prior studies in other regions [41] creating an important source of uncertainty. Elevation change data from sedimentation and erosion tables located in marshes on the Anacostia River fall in the range of those used in the model (NPS unpublished data), but it is not clear whether narrow marsh strips will behave similarly. To test the importance of accretion rate estimates, we ran the model with two modified accretion curves: one in which the higher elevation peak reached 5 mm/yr, similar to the lower peak, and the other with a single, broad peak up to 12 mm/yr (Fig 3). The first modification caused area distribution differences in 2100 of less than 1% for most classes, but a 5% increase in irregularly flooded marsh, which was offset by less land lost to open water. The second modification with the highest peak led to a result in which only the high SLR with protected development scenario showed appreciable loss of tidal habitat, emphasizing the potential for accretion to offset SLR if the suspended sediment supply rate is high enough [53, 54]. We believe the model would be similarly sensitive to reductions in estimated accretion rates leading to habitat loss.

These results emphasize two factors that contribute to marsh area change: (1) inland migration and (2) vertical maintenance vs. submergence. A third factor–shoreline erosion vs. progradation–has also been identified in previous modeling studies of marsh area change [55]. Inland migration depends on SLR rate, inland topography [22], and anthropogenic prevention of inundation (e.g., levee construction) [21]. High SLR rates and low-lying land areas will lead to rapid inland migration of the marsh edge, and if suspended sediment supplies and marsh productivity are high enough to prevent loss of pre-existing marshland, then total area will expand. Interaction among these three factors leads to four main cases: (1) marsh expansion both inland and seaward, (2) marsh expansion while migrating inland, (3) marsh contraction while migrating inland, and (4) inundation [55]. Because we do not include shoreline erosion or progradation in MAIM, due to an inability to adequately model the processes, our results are limited to the final three cases, with shoreline retreat caused by point inundation alone. In general, we see case 2 for the unprotected 0.7 m and 1.1 m SLR scenarios and a mix of case 2 and 3 for the unprotected 1.7 m SLR scenario (e.g., Figs 5 and 8). When development is protected and inland migration is prevented in most areas, we see case 3 for the 0.7 m and 1.1 m SLR scenarios, and case 4 for the highest SLR (e.g., Figs 6 and 7).

It is important to note that MAIM is not a hydrodynamic model capable of simulating spatially variable sediment dynamics. This is true for the deposition of sediment near tidal channels [56], but also the erosion of sediment from shorelines [54, 57, 58]. We know that shorelines in some areas are receding due to wave action [59], but this process is far from universal. We mapped shoreline change between 1990 and 2012 using NAIP imagery, but the rates varied unpredictably in time, and potential predictors such as fetch [60] had limited explanatory power, possibly because much of the shoreline is hardened. Although past work has emphasized the role of shoreline erosion in creating open water from tidal habitat [54], incorporating this process into a freshwater estuarine model like MAIM remains a future goal. Nonetheless, because the elevation dependent elevation change rate approaches SLR rates (particularly for the low SLR scenario), the primary constraint on habitat area is topography and development, which we model effectively.

Salinity effects are not characterized in this implementation of MAIM, under the assumption that the region will retain its freshwater characteristics through the end of the modeling period. If brackish water were to intrude into the study area we would expect habitat loss for both marshes [61] and swamps [62]. Saline water dramatically changes biogeochemical processes in a marsh, typically leading to decay of buried organic material and elevation collapse [63, 64]. According to Whitehead and colleagues [65] average salinity was very low (0.5 psu) in the study area from 1986–2005 and extending as far as Maryland Point (Table 4, Fig 1). This is near the center of the ‘hydrodynamic transition zone’ in the Potomac recognized in 1984 by Callender and colleagues [66] extending from Quantico, VA to the US Highway 301 bridge (Fig 1). This zone does not appear to be migrating rapidly, as Cornwell and colleagues [67] only saw salinity values above 1 psu beginning near Quantico during the summer low flow season in 2010 and just above the Highway 301 bridge during spring high flows in 2011 (Table 4). If we assume that deliveries of freshwater down the Potomac River will remain stable through 2100, then changes to cross sectional area of flow would be the dominant process by which SLR may decrease downriver fluxes and velocities and promote upstream salt intrusion. At present, the Potomac at Quantico has a cross sectional area of 16,000 m^2^, while the cross section at Indian Head has an area of 11,000 m^2^, and 6,000 m^2^ at Marshall Hall in Piscataway Park (in the lowermost sub-region PISC W, Fig 1). If sea levels rise 1.7 m, the highest we tested, the cross sectional areas at Indian Head and Piscataway would increase to 15,000 m^2^ and 8,000 m^2^, respectively (Table 4). These values are lower than the present cross sectional areas at Quantico and points downstream [68], suggesting that salt intrusion may not extend beyond Indian Point in our modeling scenarios, although 2-D hydrodynamic modeling would be required to test this more fully.

**Table 4:** Potomac River salinity and cross sectional areas of flow.

Location	Distance downstream of DC (km)	Salinity regime (~2010)^a^	Channel width (m)	x-section area (2010 estimate, m^2^)	x-section area (m^2^) 0.7 m SLR	x-section area (m^2^) 1.1 m SLR	x-section area (m^2^) 1.7 m SLR
US Hwy 301 Bridge	98	5–15 psu	3580	19,000^b^	-	-	-
Maryland Point	75	0.5–5 psu	2190	14,000 ^b^	-	-	-
Quantico	50	0.5–5 psu	2310	16,000	17,900	18,800	20,200
Indian Head	38	< 0.5 psu	2100	11,000	12,500	13,300	14,600
Marshall Hall	25	< 0.5 psu	6020	6,000	7,000	7,500	8,300

^a^ Salinity data from Cornwall et al. 2015

^b^ Lower river cross-sectional area values from Herman and Friedrichs, 2010

Model validation was performed on synthetic landscapes and compared with SLAMM version 6, with MAIM assumptions modified to match those of SLAMM (e.g., fewer land classes, simplified class switching structure, uniform or cubic accretion functions for each land class). Model outputs under these conditions agreed in all details. Verification of the parameters applied in this study, however, was not possible due to the lack of historical elevation data of adequate precision to permit a hindcasting exercise. Future workers may wish to verify results by analyzing the geospatial output to find locations that are predicted to switch class shortly. Field observations from these zones that are near a threshold may show signs of pending change, such as recent tree ring narrowing, enhanced flooding, soil alteration, or establishment of species associated with the anticipated future habitat class.

In our analysis there was not always a monotonic relationship between increased SLR rate and increased tidal habitat loss, because the model included elevation dependent elevation gain. In the unprotected development scenarios, we observe an optimum SLR rate where area gain is maximized, due to the tidal flooding of dry land in combination with marsh surface accretion that maintains existing marsh, analogous to the maxima modeled by Kirwan and colleagues [55] and inferred by Morris and colleagues from biomass data in a South Carolina salt marsh [69]. In contrast, protecting development always led to tidal habitat reduction. It is unlikely that maximum development loss will be accepted by tidewater communities [21], however our results highlight an ability to alleviate habitat loss in high SLR scenarios by permitting some managed open space to be tidally inundated. Soft development such as parks and golf courses can be thought of as a habitat reserve, but optimal management will need to be agile and responsive to actual SLR, given the range of possible scenarios.

## Supporting Information

S1 Fig DEM elevations vs. RTK-GPS survey elevations showing bias and systematic error in the DEM used in this analysis. RTK data from Feb 2012; 1341 points.
(TIFF)

S2 Fig Decision tree used to generate the initial vegetation classification. (TIFF)

S1 File DEM Validation. Analysis of digital elevation model accuracy in marsh areas where standing biomass inhibits laser pulses from reaching the mineral sediment surface.
(DOCX)

S2 File Mapping procedure. The initial land classification map was based on a combination of elevation data, water and forest mapping from aerial photographs, and pre-existing vegetation community maps.
(DOCX)

S3 File Initial Map Validation. The initial land classification maps were compared against 246 field validation points collected in three sub-areas with diverse habitat classes and subdued elevation variation.
(DOCX)

S1 Table Field validation outcome of initial classification map (DOCX)

S2 Table Differences between initial classification map and map adjusted for MAIM elevation assumptions (t_0_ adjustment) (DOCX)
