# Supplementary figures and images for: Modeled Tradeoffs between Developed Land Protection and Tidal Habitat Maintenance during Rising Sea Levels
Source: PLoS One. 2016 Oct 27;11(10):e0164875. doi: 10.1371/journal.pone.0164875 (PMC5082943; doi:10.1371/journal.pone.0164875)

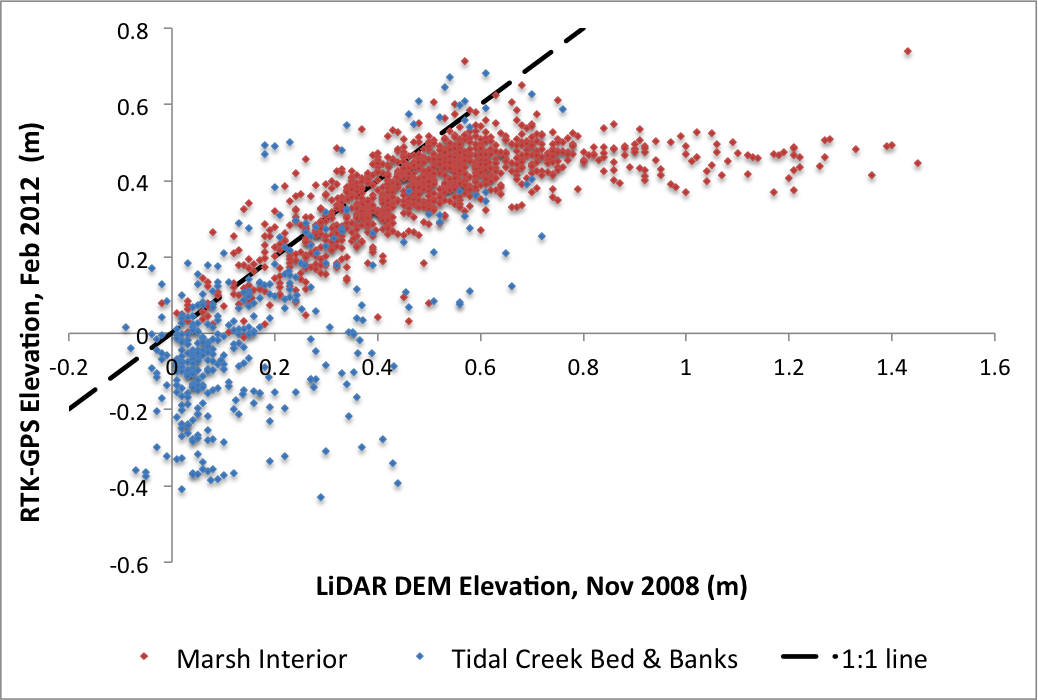

Supplement: S1 Fig — RTK data from Feb 2012; 1341 points. (TIFF) [file pone.0164875.s001.tiff]

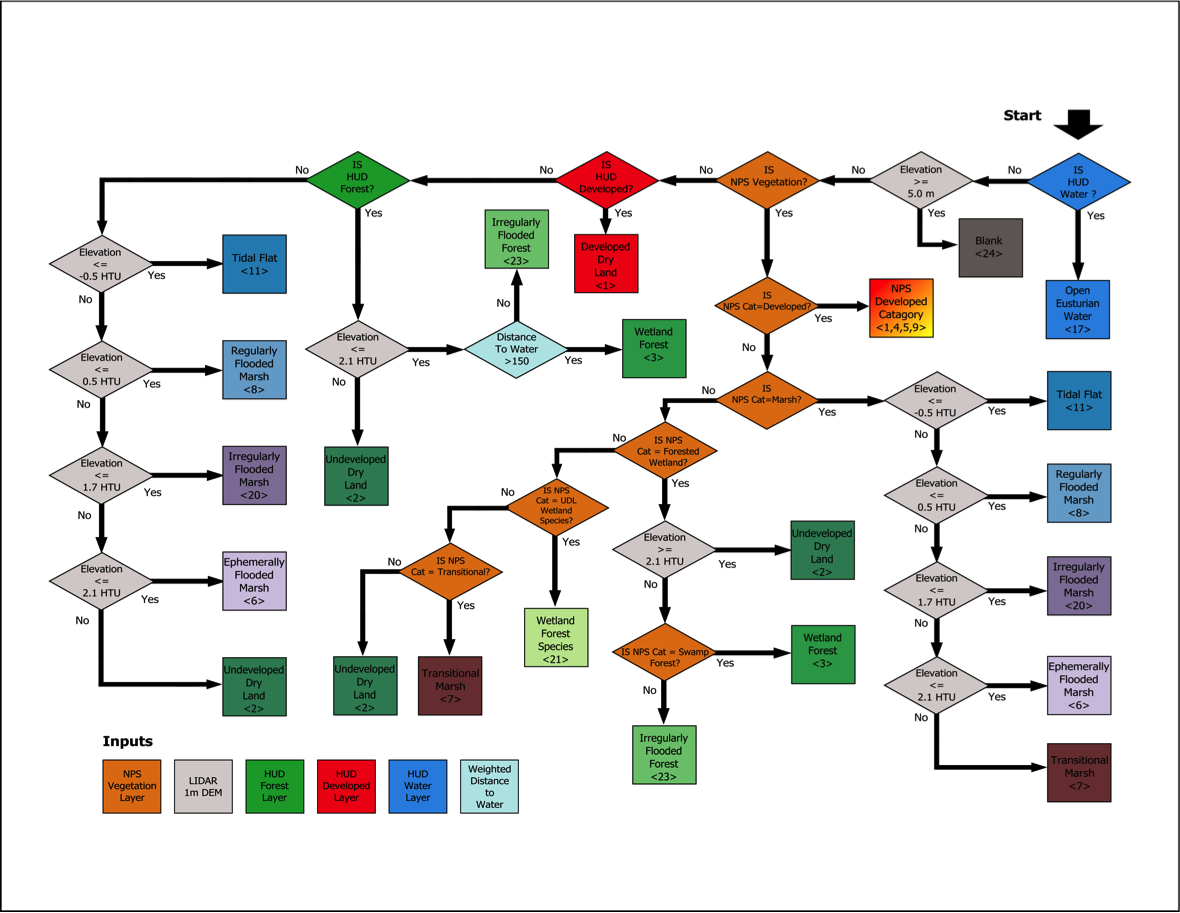

Supplement: S2 Fig — (TIFF) [file pone.0164875.s002.tiff]
