# Supplementary material for: Modeled Tradeoffs between Developed Land Protection and Tidal Habitat Maintenance during Rising Sea Levels
Source: PLoS One. 2016 Oct 27;11(10):e0164875. doi: 10.1371/journal.pone.0164875 (PMC5082943; doi:10.1371/journal.pone.0164875)
Supplement: S1 File — Analysis of digital elevation model accuracy in marsh areas where standing biomass inhibits laser pulses from reaching the mineral sediment surface. (DOCX) [file pone.0164875.s003.docx]

**Supporting Section S1: DEM Validation**

The accuracy of the DEM was checked using real time kinematic (RTK) global positioning system (GPS) survey points. Hard surfaces, such as roads, were very accurately represented by the DEM (~ 2 cm root mean squared error), so the majority of our validation work focused on difficult terrains, in particular marshes. Vegetation shields the ground surface from LiDAR detection, making characterization of the ground surface more difficult. Forests and marshes create different challenges. Forests have taller vegetation, so the difference between canopy returns and ground returns is easier to distinguish. Dense marsh vegetation may block any true ground over very large areas, interfering with ground filtering algorithms. Additionally, the returns from the low canopy may be mistaken for ground returns.

Plotting RTK-GPS elevation data against the DEM elevation at the same locations enabled us to identify systematic errors and bias in the DEM. To this end we collected an extensive survey data set in Dyke Marsh Wildlife Preserve. At elevations representative of the low marsh vegetation community, which has shorter individuals and more gaps between foliage, survey and DEM elevations were in general agreement with a slight positive bias in the DEM (Figure S1). In elevations representative of the high marsh vegetation community, the average positive error in the DEM increased with height and the range of uncertainty increased greatly with height (Figure S1). Points with survey elevations between 0.4 and 0.5 m had DEM elevations ranging from 0.4 to 1.4 m. This is presumably due to the variable depth of penetration of the LiDAR pulses into the marsh canopy.
