# Supplementary material for: Modeled Tradeoffs between Developed Land Protection and Tidal Habitat Maintenance during Rising Sea Levels
Source: PLoS One. 2016 Oct 27;11(10):e0164875. doi: 10.1371/journal.pone.0164875 (PMC5082943; doi:10.1371/journal.pone.0164875)
Supplement: S2 File — The initial land classification map was based on a combination of elevation data, water and forest mapping from aerial photographs, and pre-existing vegetation community maps. (DOCX) [file pone.0164875.s004.docx]

**Supporting Section S2: Mapping Procedure**

The NPS provided a vector file consisting of non-overlapping polygons classified by vegetation community. The NPS maps were based on aerial photography digitalization followed by vegetation community inventories at 425 plots and validation at 1895 rapid assessment locations (Hazler et al., 2012). Forest structure was finely classified, while marsh structure tended to be more coarsely delineated. To supplement this data set, forest areas were manually digitized from 2009 Near Infra-Red (NIR) NAIP aerial photographic imagery at 1 m resolution. This exercise included all modeled lands and therefore filled gaps in the NPS vegetation layer occurring in areas outside NPS lands. All NAIP imagery was acquired during the summer growing season (leaf-on) and under clear-sky conditions. We compared our digitized forest maps with the NPS maps and where the newly digitized maps exhibited greater detail and positional accuracy along forest boundaries we used the newly created forest layer in place of the NPS vegetation maps. In cases where only the boundaries differed, we joined NPS map attributes to our forest layer so that these attributes could be used in the initial classification tree.

Based on field surveys (Cadol et al., 2014) it was found that elevation thresholds adequately delineated the boundaries between several important land cover types. The elevation 0.5 HTU was highly successful at separating Ephemerally Flooded Marsh from Irregularly Flooded Marsh. We used 1.7 HTU as an upper limit to Irregularly Flooded Marsh because in areas with accurate elevation data in the DEM this was the limit of strong tidal influence. There were some areas within active tidal marshes, however, where the DEM bias was quite strong. The standing vegetation reflected the LiDAR laser pulses and caused the DEM surface to be much higher than the actual ground surface. In these cases our classification scheme assigns the area to the Ephemerally Flooded Marsh or Transitional Scrub categories.

A weighted distance to water measure was calculated to differentiate between Irregularly Flooded Forest, where there is a strong tidal influence, and Wetland Forest, which are potentially as wet as Irregularly flooded forest, but where there is minimal tidal influence. The two forest types might contain the same forest species, but different hydrologic processes influence the habitat. To represent this difference we generated a cost-distance to water, which was meant to reflect the potential for tidal water to reach these forests. The cost-distance was calculated for each grid cell as the distance to the nearest shoreline weighted by the elevation squared, and is therefore a unit-less number. The threshold value of weighted distance from Estuarine Open Water used in classifying Ephemerally Flooded Forest and Wetland Forest was found separately for each analysis area by inspecting histograms of weight distance values for areas classified as tidal swamp and swamp forest in the NPS vegetation maps. Typically there was good clustering and a threshold value was clear. The best threshold was considered to be the one for which an equal percentage of misclassification occurred for each of the two forest types. A threshold of 150 was acceptable for all but one site, where the threshold was set at 400. Forests closer to tidal water than this threshold distance were mapped as Irregularly Flooded Forest and forests more distant from water were mapped as Wetland Forest. Although we relied on the NPS classification polygons to set a threshold, we were not comfortable using the polygon areas directly in our classification system due to the coarseness of the mapping. For example, there were often small areas within larger upland forest polygons that our system was able to identify as likely swamps due to the locally low elevation.

All of these data sets were combined into a classification scheme, depicted in Figure S2. Transitional Scrub and the various marsh categories graded into one another, with inundation characteristics ranging from extremely rare (but with expected hydrological influence from the tides) to daily for the majority of the day. Tidal Flats may be vegetated with submerged aquatic vegetation (SAV) or may be barren mud flat, but the DEM available in our study area could not identify these elevations due to submergence during LiDAR flights, so this habitat was hand digitized using NAIP imagery. The outer edges of these zones were assigned an elevation of -1.3 HTU (the mean lower elevation limit of this category) with the rest of the elevations interpolated linearly to the edge of Regularly Flooded Marsh. Estuarine Open Water shared this elevation limit of -1.3 HTU but had no lower limit.
