# Supplementary material for: Modeled Tradeoffs between Developed Land Protection and Tidal Habitat Maintenance during Rising Sea Levels
Source: PLoS One. 2016 Oct 27;11(10):e0164875. doi: 10.1371/journal.pone.0164875 (PMC5082943; doi:10.1371/journal.pone.0164875)
Supplement: S3 File — The initial land classification maps were compared against 246 field validation points collected in three sub-areas with diverse habitat classes and subdued elevation variation. (DOCX) [file pone.0164875.s005.docx]

**Supporting Section S3: Initial Map Validation**

For verification, the classification maps were compared to point habitat classifications collected in the field. Transects were walked through areas with variable habitat, and classification points were collected both within large habitat patches as well as at boundaries between patches, creating a rigorous verification data set. Marsh and tidal flat habitats were adequately described by the classification procedure (Table S1). We were unable to collect adequate data to distinguish tidal forest and wetland forest classifications, but the lower edge of the tidal forest was satisfactorily demarcated. Transitional Scrub areas were not fully verified due to the diversity of situations this class includes. These initial classes were primarily identified from the NPS classification polygons, and there was little consistency in terms of elevation or distance from water for the distribution of these areas. Rather, land use history seems to be the dominant control on the establishment of transitional vegetation, as we have defined it. At issue may be the very concept of a transitional zone within a tidal freshwater setting, as opposed to a salt marsh setting.
