# Supplementary material for: Modeled Tradeoffs between Developed Land Protection and Tidal Habitat Maintenance during Rising Sea Levels
Source: PLoS One. 2016 Oct 27;11(10):e0164875. doi: 10.1371/journal.pone.0164875 (PMC5082943; doi:10.1371/journal.pone.0164875)
Supplement: S1 Table — (DOCX) [file pone.0164875.s006.docx]

**Table S1.** Field validation of initial classification map

|  | Little Hunting Creek | | Piscataway | | Southern Dyke Marsh | | Total | | |
| --- | --- | --- | --- | --- | --- | --- | --- | --- | --- |
|  | n = 158 | | n = 45 | | n = 43 | | n = 246 | | |
|  | # correct | % correct | # correct | % correct | # correct | % correct | | # correct | % correct |
| All types exact ^a^ | 101 | 64% | 18 | 40% | 39 | 91% | | 158 | 64% |
| IM+EM exact ^b^ | 110 | 70% | 29 | 64% | 39 | 91% | | 178 | 72% |
| All types w/in 1 m ^c^ | 116 | 73% | 20 | 44% | 40 | 93% | | 176 | 72% |
| IM+EM w/in 1 m ^d^ | 122 | 77% | 31 | 69% | 40 | 93% | | 193 | 78% |

^a^ All types exact: Number/percent of collected points that were correctly classified in the initial classification map as tidal flats, regularly flooded marsh, irregularly flooded marsh, ephemerally flooded marsh, transitional scrub, or tidal swamp.

^b^ IM+EM exact: Same as all types exact, except that the irregularly flooded marsh and ephemerally flooded marsh were combined into a single category

^c^ All types w/in 1 m: Number/percent of collected points that were within 1 meter of a cell of the same classification in the initial classification map,

^d^ IM+EM w/in 1 m: Same as all types w/in 1 m, except that the irregularly flooded marsh and ephemerally flooded marsh were combined into a single category.
