# Supplementary material for: Modeled Tradeoffs between Developed Land Protection and Tidal Habitat Maintenance during Rising Sea Levels
Source: PLoS One. 2016 Oct 27;11(10):e0164875. doi: 10.1371/journal.pone.0164875 (PMC5082943; doi:10.1371/journal.pone.0164875)
Supplement: S2 Table — (DOCX) [file pone.0164875.s007.docx]

**Table S2.** Model changes to initial land classification to conform to class elevation limits

| Land Class | Area in initial classification (ha) | Area after elevation correction (ha) | Change in area (ha) | Fraction change in area |
| --- | --- | --- | --- | --- |
| Developed Dry Land (non-NPS) | 717 | 717 | 0 | 0.00 |
| Developed Dry Land (NPS) | 176 | 176 | 0 | 0.00 |
| Managed Open Space | 328 | 328 | 0 | 0.00 |
| Agriculture | 93 | 93 | 0 | 0.00 |
| Undeveloped Dry Land | 556 | 555 | -1 | 0.00 |
| Undeveloped Dry Land with Swamp Species | 163 | 158 | -5 | -0.03 |
| Wetland Forest | 37 | 76 | 39 | 1.05 |
| Irregularly Flooded Forest | 190 | 119 | -71 | -0.37 |
| Transitional Scrub | 58 | 59 | 1 | 0.02 |
| Ephemerally Flooded Marsh | 30 | 38 | 8 | 0.27 |
| Irregularly Flooded Marsh | 128 | 179 | 51 | 0.40 |
| Regularly Flooded Marsh | 147 | 160 | 13 | 0.09 |
| Tidal Flat | 197 | 162 | -35 | -0.18 |
| Estuarine Open Water | 4105 | 4105 | 0 | 0.00 |
